# Hand–eye calibration for rigid laparoscopes using an invariant point

**DOI:** 10.1007/s11548-016-1364-9

**Published:** 2016-03-19

**Authors:** Stephen Thompson, Danail Stoyanov, Crispin Schneider, Kurinchi Gurusamy, Sébastien Ourselin, Brian Davidson, David Hawkes, Matthew J. Clarkson

**Affiliations:** Centre for Medical Image Computing, Front Engineering Building, University College London, Malet Place, London, UK; Division of Surgery, Hampstead Campus, UCL Medical School, Royal Free Hospital, 9th Floor, Rowland Hill Street, London, UK

**Keywords:** Hand–eye calibration, Laparoscope, Tracking, Augmented reality

## Abstract

**Purpose:**

Laparoscopic liver resection has significant advantages over open surgery due to less patient trauma and faster recovery times, yet it can be difficult due to the restricted field of view and lack of haptic feedback. Image guidance provides a potential solution but one current challenge is in accurate “hand–eye” calibration, which determines the position and orientation of the laparoscope camera relative to the tracking markers.

**Methods:**

In this paper, we propose a simple and clinically feasible calibration method based on a single invariant point. The method requires no additional hardware, can be constructed by theatre staff during surgical setup, requires minimal image processing and can be visualised in real time. Real-time visualisation allows the surgical team to assess the calibration accuracy before use in surgery. In addition, in the laboratory, we have developed a laparoscope with an electromagnetic tracking sensor attached to the camera end and an optical tracking marker attached to the distal end. This enables a comparison of tracking performance.

**Results:**

We have evaluated our method in the laboratory and compared it to two widely used methods, “Tsai’s method” and “direct” calibration. The new method is of comparable accuracy to existing methods, and we show RMS projected error due to calibration of 1.95 mm for optical tracking and 0.85 mm for EM tracking, versus 4.13 and 1.00 mm respectively, using existing methods. The new method has also been shown to be workable under sterile conditions in the operating room.

**Conclusion:**

We have proposed a new method of hand–eye calibration, based on a single invariant point. Initial experience has shown that the method provides visual feedback, satisfactory accuracy and can be performed during surgery. We also show that an EM sensor placed near the camera would provide significantly improved image overlay accuracy.

## Introduction

The successful implementation of an image guidance system for laparoscopic liver resection has the potential to improve the feasibility of laparoscopic resection for patients with tumours located in surgically challenging locations. If done well, laparoscopic resection can have equivalent curative results to open surgery but with shorter recovery times [16]. However, an accurate vision system for registration and reconstruction requires precise calibration. The calibration process determines the camera intrinsic parameters [28], and when an external tracking device is used, the calibration process also determines the precise position and orientation of the tracking markers relative to the camera coordinate system. This second process, is known as “hand–eye” calibration, a term that originates from the robotics literature [13, 27].

The most commonly suggested form of image guidance is to project information from pre-operative data such as computed tomography (CT) or magnetic resonance (MR) scans on top of the laparoscopic video [11, 25]. This requires very precise tracking and hand–eye calibration due to the small field of view and high level of magnification. The problem is exacerbated as the tracking markers are placed on the distal end of the laparoscope with the camera on the proximal end, producing a lever effect. In addition, the surgical environment itself presents difficulties, as tracking markers must normally be attached under sterile conditions, by clinical staff, without disrupting the surgical workflow.

So, while hand–eye calibration is widely and routinely performed in robotics, the specific requirements of laparoscopic surgery mean that hand–eye calibration is still considered an unsolved problem. Current commercial systems for laparoscopic liver surgery such as those by Cascination^1^ and Pathfinder^2^ avoid the problem by displaying pre-operative data next to the laparoscopic video, rather than as an augmented reality overlay. The “SmartLiver” system under development by ourselves [25] avoids the need for precise hand–eye calibration by using the camera for both localisation and visualisation of the liver, so errors in the estimation of the hand–eye transform have a lesser effect on overlay accuracy than if the liver were localised with a second tracked object. To date, the calibration method presented here has been used during image guidance on eight patients. Calibration can be performed in around 3 min without compromising sterility. The method is sufficiently accurate to enable overall system errors of 2.9 mm when measured on a static phantom, see [25].

Therefore, in this paper we survey the literature, propose a simple method that we have used during surgery, compare our method with the most common existing methods, evaluate the performance of such calibration methods using two types of tracker (electromagnetic and optical) and discuss the steps forward.

### Background

There is a broad range of camera calibration literature, derived from fields such as photogrammetry and computer vision. Within medical applications, intrinsic parameters have been derived from the projection matrix [14], or via nonlinear optimisation [26]. While some authors use a 3D shape of known geometry [7], most [15, 21, 26] adopt a 2D pattern due to ease of manufacturing. Zhang’s work [28] has become widely used due to popular open-source implementations within MATLAB^3^ or OpenCV^4^[2]. While there is some evidence that circle or dot patterns can be more accurately located than chessboard patterns [6], and tag-based patterns[18] can be used to cope with partial views of a calibration board, the chessboard pattern remains the most prevalent in medical applications [13, 25].

Hand–eye calibration has been widely studied within the robotics literature [20]. Early, linear solutions for hand–eye calibration solved rotation and translation parameters separately in the rotation group [22, 27] or using quaternions [3], but estimates of the translational part are affected by errors in the rotational part. Linear methods to estimate both the rotation and translation simultaneously have been proposed using dual quaternions [5] and may be followed by nonlinear optimisation [8], or global optimisation [9]. It has also been shown that camera intrinsic calibration is not independent from hand–eye calibration [10].

However, within medical applications, methods for calibration must be compatible with sterility constraints, and not interrupt the surgical workflow. If a calibration object is tracked, such that chessboard corner locations are known in the reference frame of the tracking system, the hand–eye method can be solved directly using Procrustes analysis [1]. This provides a simple solution, requiring just a single view of the laparoscope tracking marker, and the tracked calibration object [7, 12, 14, 15, 17, 21], although it is possible to sample many frames and take an average to reduce the effects of noise [17].

Recent interest in the medical domain suggests that the problem is not yet solved. Malti and Barreto describe a system based on the minimum of three views with two independent rotations [13] and optimise hand–eye, intrinsic and grid-to-world transformation simultaneously by minimising the projection error of chessboard corners. Kang et al. [11] calibrate a stereo laparoscope with very narrow baseline by using a tracked pointer to register chessboard corners to the tracking system. They do this independently for right and left channels.

### Contribution of this paper

This paper describes an invariant point approach to hand–eye calibration. The system is of particular use when using laparoscopes with fixed optical properties, where due to the attachment of tracking markers in surgery, only hand–eye calibration is required. The algorithm is implemented as part of the NifTK [4] software package, while the hardware can be easily made in theatre. The main focus of the method has been developing an intuitive, fast and easy-to-use method that can be performed and validated by theatre staff. The system meets these design goals and has been used on our last eight patients. In this paper, we show that the algorithm is also capable of accurate and repeatable hand–eye calibration, with results at least as good as existing approaches.

**Fig. 1:**
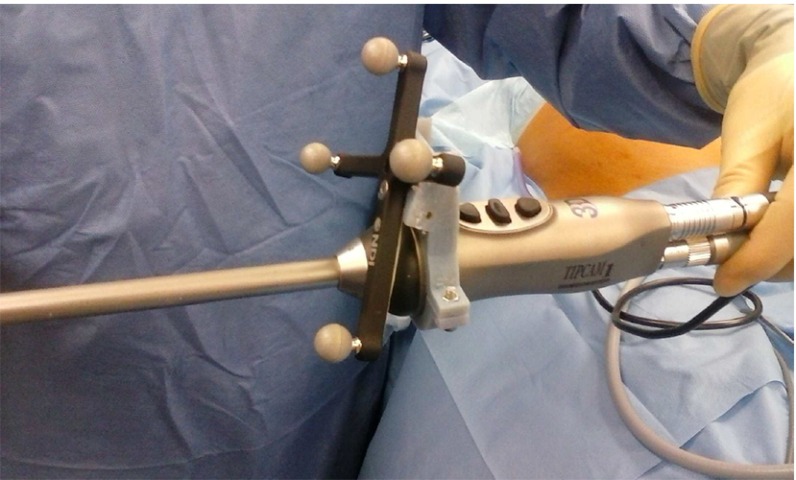
3D printed tracking collar used in surgery prior to covering in a sterile sheath

**Fig. 2:**
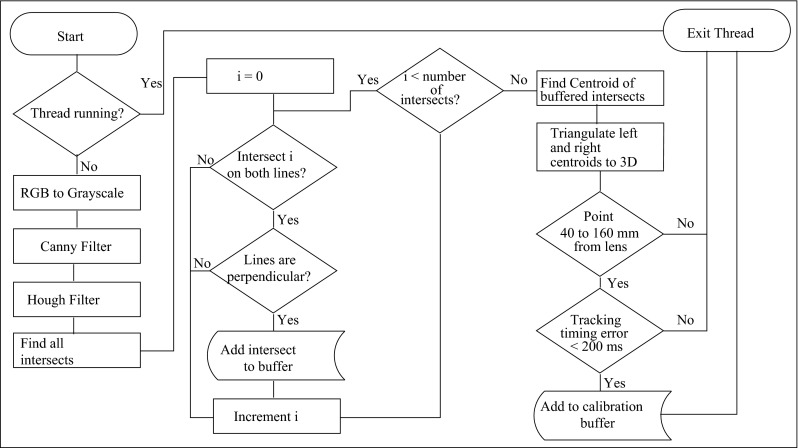
Flow chart of the image processing and acquisition routine

The paper also contributes a comparison of laparoscope tracking using an optical (NDI Polaris Spectra) and an electromagnetic (NDI Aurora) tracking system. Laparoscopes are uniquely difficult to track with optical systems, as the tracking markers must be placed on the external part of the laparoscope, far from the lens, magnifying the effect of tracking errors. Tracking with an EM marker placed near the lens can avoid this problem.

## Methods

### Set-up in theatre

The Polaris tracking cameras are first positioned within the operating theatre, in a location to maximise visibility of the tracking markers throughout surgery. We attach a 3D printed tracking collar to the laparoscope, as shown in Fig. 1. The tracking collar is designed to enable different attachment orientations that maximise tracker visibility for a range in theatre situations. At present, the tracking collar is not sterile, so a transparent sterile sheath is pulled over the collar after attachment. In the longer term, we intend to manufacture a sterile tracking collar. A sterile, single-use, crosshair is made by drawing with a sterile marker on sterile paper. The size of the crosshair is approximately 25 mm, though the exact size is not critical. As the crosshair is sterile, it can be placed on a rigid surface near the centre of the tracking camera’s operating volume without contaminating the patient or the laparoscope.

### Data acquisition

The camera calibration user interface is opened, and intrinsic calibration parameters are set. The system relies on the laparoscope being of a fixed (or at least controlled) focal length, so the intrinsic parameters do not change significantly over timer. Therefore, full calibration of the camera intrinsic parameters can be done periodically outside of surgery. The laparoscope is moved into position to image the cross. The intention is that only the cross should be visible on a plain background, and then, the start acquire button pressed.

During acquisition, a background thread runs continuously to process the crosshair images. We deliberately do not buffer images, and the intent is not to capture every frame. In general, the system processes up to five frames a second, which provides an intuitive user interface. Figure 2 shows a flow chart of the image processing process.

Provided the user maintains good camera position the calibration buffer progress bar will steadily fill. The image processing pipeline has proved robust to false-positive identification of the crosshair centre. It is up the user to ensure that the data acquisition covers a representative range of views. The user should aim to cover as much of the laparoscope’s tracked volume as possible. With an optical tracker, we find that this results in 60$^{\circ }$ pyramid with its apex at the crosshair centre. Moving the laparoscope steadily through this volume typically results in approximately 130 frames of data in the calibration buffer. At the end of data acquisition, the user presses stop acquire and then starts calibration optimisation.

### Proposed invariant point calibration

The calibration data buffer now contains a vector of measured on-screen crosshair locations, $X_\mathrm{MS}$, of length 2*n* (for a stereo laparoscope), and a vector, $T_\mathrm{marker}$, of length *n*, of corresponding tracking matrices for the tracked markers. Capital letters are used to refer to vectors while lower-case letters refer to individual elements.

**Fig. 3:**
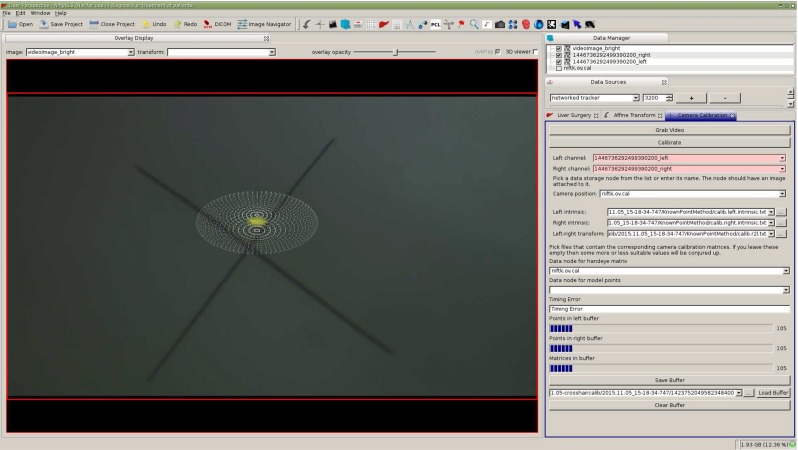
Image of the crosshair with a 10-mm-diameter sphere overlaid. As the user the moves the laparoscope, the visible crosshair should stay within the bounds of the sphere. The user can set the diameter of the sphere to their requirements

ITK’s implementation of the Levenberg–Marquardt least squares optimiser is used to find optimal values for the six parameters of the hand–eye transform, $t_\mathrm{handeye}$, (three translations and three rotations) and, optionally, three parameters for the position of the invariant point $x_\mathrm{IP}$. The user can either measure the location of $x_\mathrm{IP}$ with a tracked pointer and fix it in the calibration, or allow the optimiser to determine it. The optimal parameters are defined as those that minimise the sum of squares of a vector of residual values, *E*, defined below in cost function Eqs. (1) and (3).

#### Cost function 1: point spread of triangulated points

The previously determined camera intrinsic calibrations and the right to left lens transform are used to triangulate $X_\mathrm{MS}$ to a vector, $X_\mathrm{LL}$, of length *n*, of 3D points in the coordinates of the left lens. For a given estimate of the hand–eye calibration, $t_{\mathrm{handeye}_{i}}$, and location of the invariant point, $x_{\mathrm{IP}_i}$, the vector, *E*, of length 3*n*, of residual values is calculated using Eq. (1).

1 $$\begin{aligned} E_j = {X_{\mathrm{world}_i,j} - x_{\mathrm{IP}_i,j}} \quad (\text {for}\quad j=x,y,z) \end{aligned}$$

where

2 $$\begin{aligned} X_{\mathrm{world}_i} = T_\mathrm{marker} \times t_{\mathrm{handeye}_{i}} \times X_\mathrm{LL} \end{aligned}$$

#### Cost function 2: projected error

The inverse of Eq. (2) is used to transform $x_{\mathrm{IP}_i}$ to a vector of 3D points in the coordinates of the left lens. These points are projected onto the left and right screens using the previously determined camera intrinsic and right to left lens calibrations, giving a vector, $X_\mathrm{PS}$, of length 2*n*, of projected points. The vector, *E*, of length 4*n*, of residual values is calculated using Eq. (3).

3 $$\begin{aligned} E_j = X_{\mathrm{PS}_j} - X_{\mathrm{MS}_j} \quad (\text {for}\,\,j=x,y) \end{aligned}$$

The optimisation process runs to convergence and alerts the user on completion. Optimisation using cost function 1 is nearly instantaneous and insensitive to initialisation. Optimisation using cost function 2 can be slow to converge and requires good initialisation. In practice, optimisation is first performed using cost function 1, initialised with the identity transform for $t_\mathrm{handeye}$ and either the origin or the measured point position for $x_\mathrm{IP}$. The output parameters may then be used to initialise optimisation using cost function 2.

### Visual validation

Upon completion of calibration, the user interface enables the immediate visualisation of the calibration result. By visualising the invariant point as a wire frame sphere with a known radius, it is possible to validate the accuracy of the calibration by moving the laparoscope around and checking whether the visible crosshair centre moves beyond the sphere boundary, as shown in Fig. 3. The ability to very quickly and intuitively evaluate the hand–eye calibration is a key benefit of this method.

## Experiments and results

### Method

**Fig. 4:**
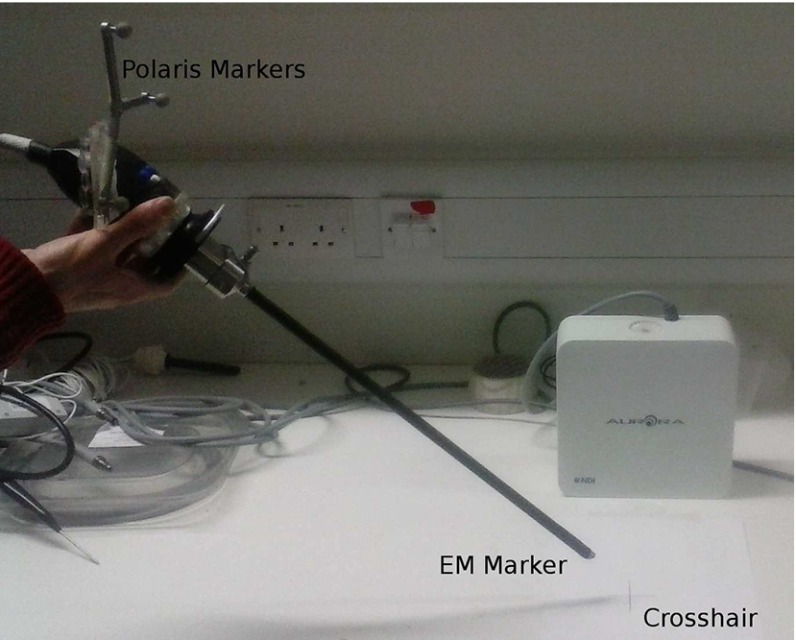
Viking 3DHD (www.conmed.com) stereo laparoscope used for testing of the calibration, showing the optical and electromagnetic tracking markers

The proposed calibration method was used to generate calibrations using ten independent data acquisitions. In each case, both cost functions (3D reconstruction error and projected error) were evaluated. The projected error optimisations were initialised with the results of the reconstruction error evaluations. In one set of experiments, both the hand–eye transform and invariant point location were optimised; in another, the position of the invariant point was measured independently using a tracked pointer and then fixed during the optimisation. All experiments were performed with an optical tracker marker attached to the distal end of the laparoscope and an EM sensor attached to the proximal end, as shown in Fig. 4. For each of the ten calibration data set, there are therefore eight calibration results.

**Fig. 5:**
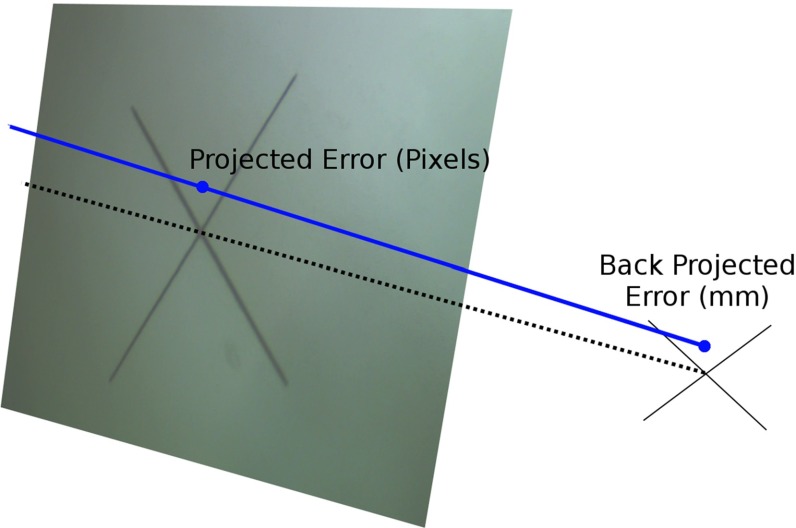
Various calibrations are used to project the measured crosshair centre back onto the screen. The resulting projected error in pixels is back projected onto the plane passing through the crosshair centre, and parallel to the image plane, to give an error in mm

As a comparison, calibration was also performed using a stationary chessboard. OpenCV was used to extract the chessboard corners and perform an intrinsic calibration of each camera channel. Two methods were used to perform the hand–eye calibration. The first was Tsai’s method ([27]); the second, “direct”, method was to measure the location of the chessboard corners with a tracked pointer and solve for the hand–eye transform directly from a stationary camera position [21].

Each method uses a different number of frames. When using Tsai’s method, we limited the number of frames to 30, to maintain a reasonable computation time. The direct method uses a single frame. Our proposed invariant point method uses around 130 frames. As there is negligible computational penalty to including more frames, we avoid the need to sub-sample the available video frames. In theory, the method would work the same with a smaller number of frames. The key requirement with both our method and Tsai’s is that the frames used are spread evenly across the tracked volume, which we maintained in both cases.

**Fig. 6:**
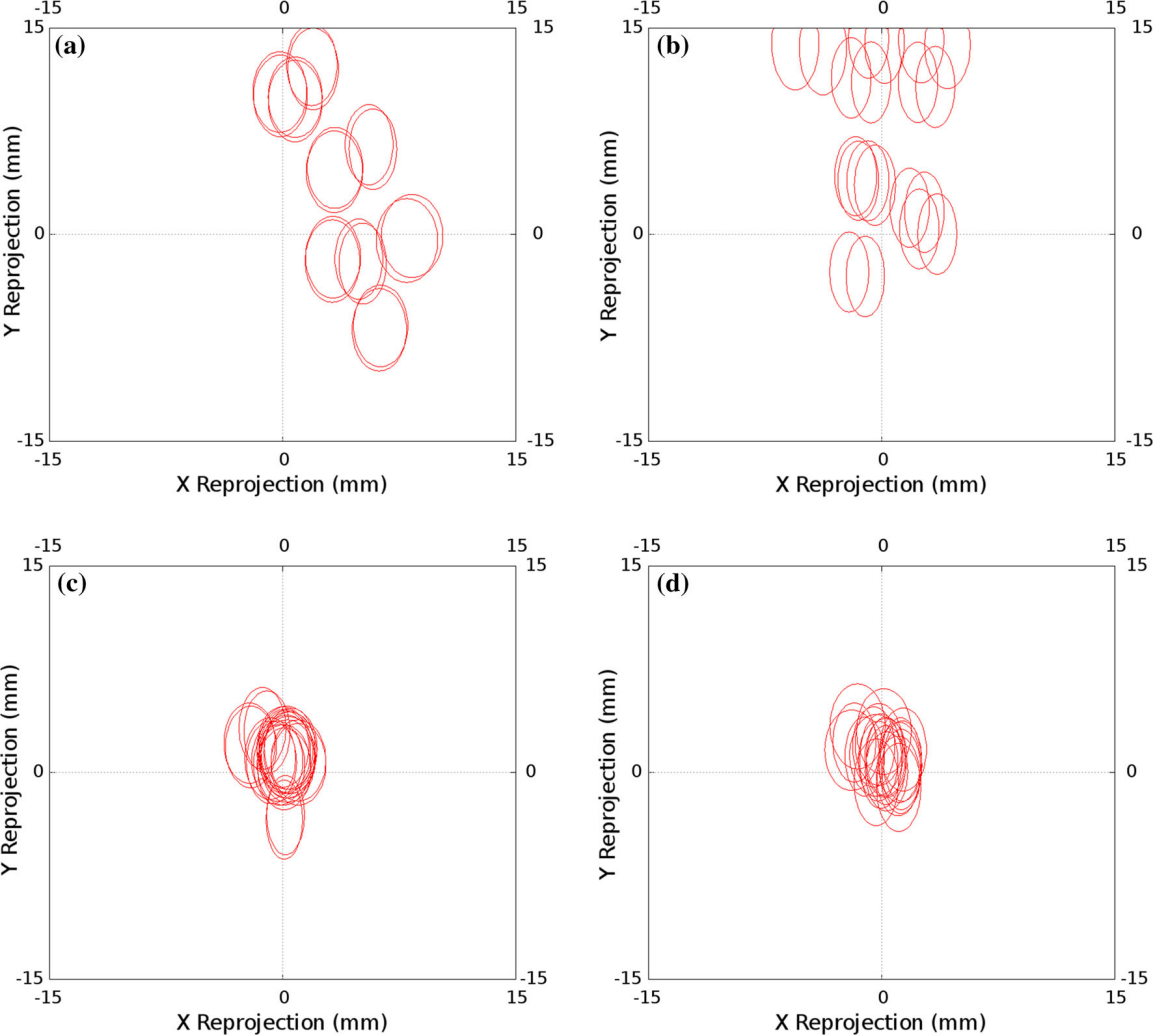
Invariant point calibration: the projection errors for each of the ten calibrations performed with the optical tracking system; two cost functions were used and two methods of finding the invariant point. The size of the individual ellipses is principally a measure of the tracking accuracy, and not greatly effected by the calibration used. The distance of the ellipse centre from the origin is the main measure of the calibration accuracy. **a** 3D error, optimised invariant point; Ellipse Centre RMS = 13.46, Ellipse Mean Radii = 4.76. **b** Projected error, optimised invariant point; Ellipse Centre RMS = 10.90, Ellipse Mean Radii = 4.35. **c** 3D error, fixed invariant point; Ellipse Centre RMS = 1.95, Ellipse Mean Radii = 4.61. **d** Projected error, fixed invariant point; Ellipse Centre RMS = 1.80 Ellipse Mean Radii = 4.42

### Experimental validation

As the true hand–eye calibration is unknown, the performance of each calibration was assessed by measuring the projection errors for a single known point. A further, independent data set imaging the crosshair was captured and the position of the crosshair measured using a tracked pointer. The on-screen crosshair centre locations were measured and compared with the on-screen locations as projected using the appropriate hand–eye result. As the projected errors in pixels are of little practical use, these errors were back projected onto the plane of the cross to give an error in mm. Figure 5 shows this process.

### Results

**Fig. 7:**
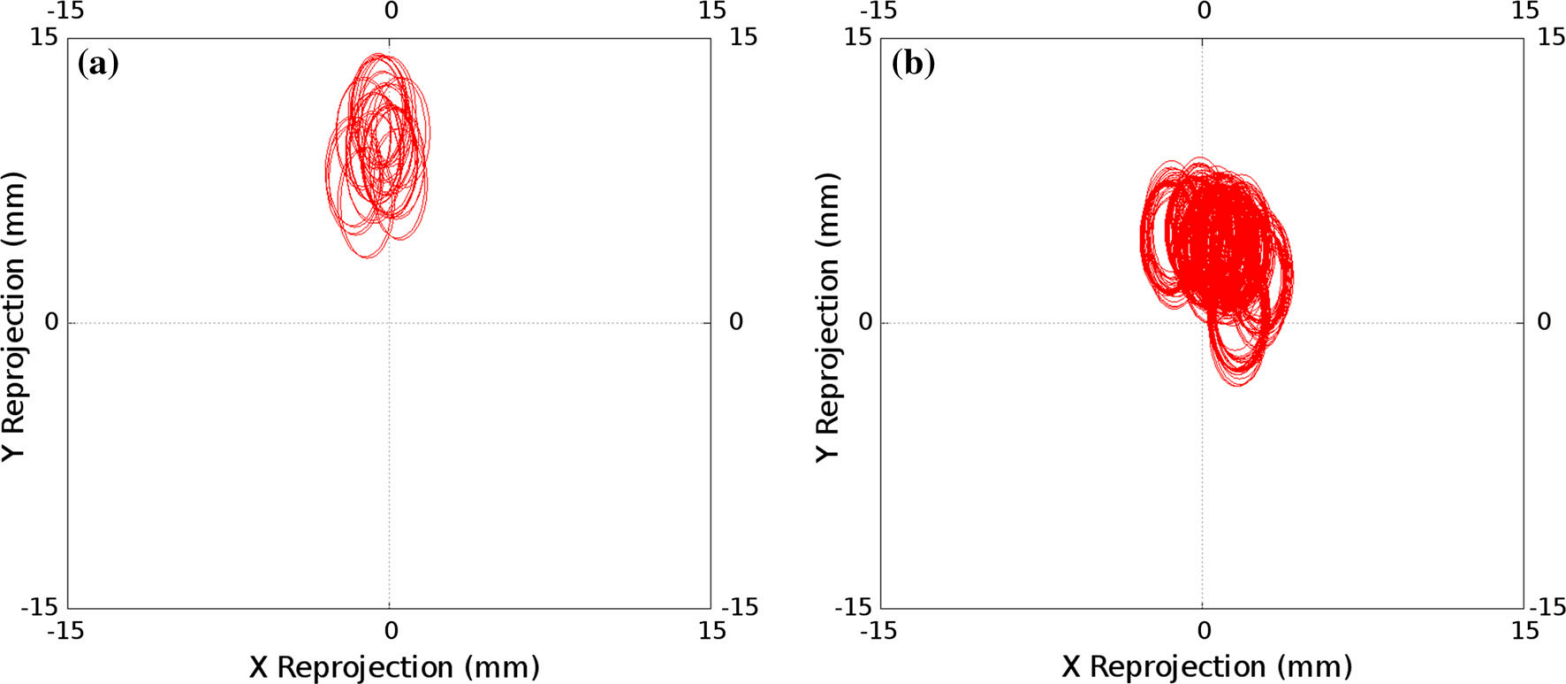
Projection errors for the calibrations performed using the chessboard using optical tracking. As in Fig. 6, the distance of the ellipse centre from the origin is the main measure of the calibration accuracy. **a** Calibration using 30 chessboard images, with hand–eye calibration performed as per Tsai’s method; Ellipse Centre RMS = 9.38, Ellipse Mean Radii = 4.15. **b** Direct calibration, using a single image of a chessboard with known corner locations; Ellipse Centre RMS = 4.13, Ellipse Mean Radii = 4.15

Figure 6 shows the results of accuracy analysis of each of the invariant point calibration algorithms applied to the optically tracked laparoscope. Each ellipse represents one standard deviation of the projection errors over the evaluation data set. The size of the individual ellipses is principally a measure of the tracking accuracy, and not greatly effected by the calibration used. The distance of the ellipse centre from the origin is the main measure of the calibration accuracy. Using projected errors gives a slightly better result than 3D errors; the most significant difference is made by independently locating the invariant point, removing it from the optimisation. Figure 7 shows the accuracy results using the chessboard calibration methods, both Tsai’s method and a direct method.

**Fig. 8:**
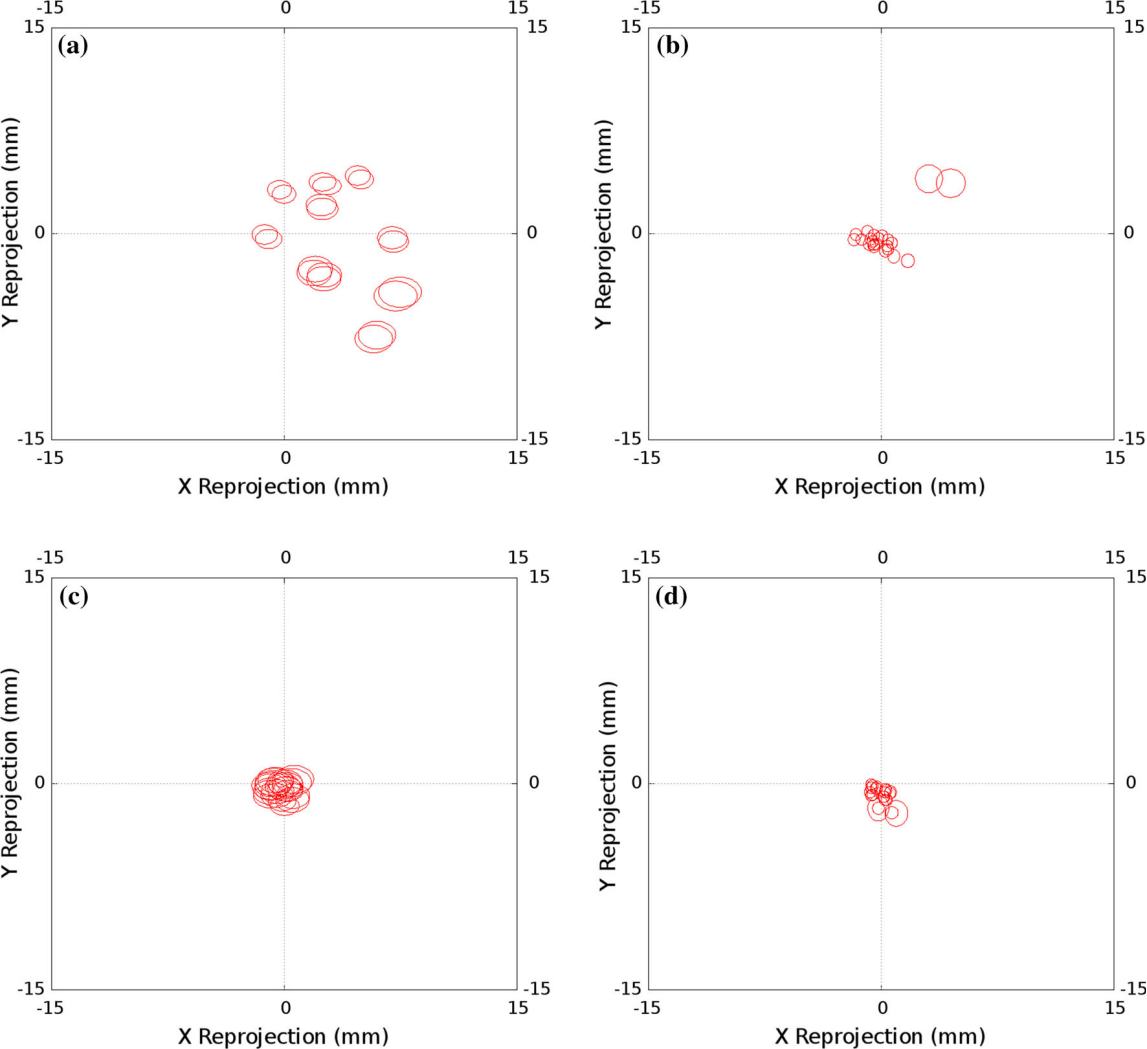
Invariant Point Calibration: the projection errors for each of the 10 calibrations performed with the electromagnetic tracking system; two cost functions were used and two methods of finding the invariant point. **a** 3D error, optimised invariant point; Ellipse Centre RMS = 8.38, Ellipse Mean Radii = 1.87. **b** Projected error, optimised invariant point; Ellipse Centre RMS = 2.00, Ellipse Mean Radii = 0.91. **c** 3D error, fixed invariant point; Ellipse Centre RMS = 0.85, Ellipse Mean Radii = 1.97. **d** Projected error, fixed invariant point; Ellipse Centre RMS = 1.14, Ellipse Mean Radii = 0.87

**Fig. 9:**
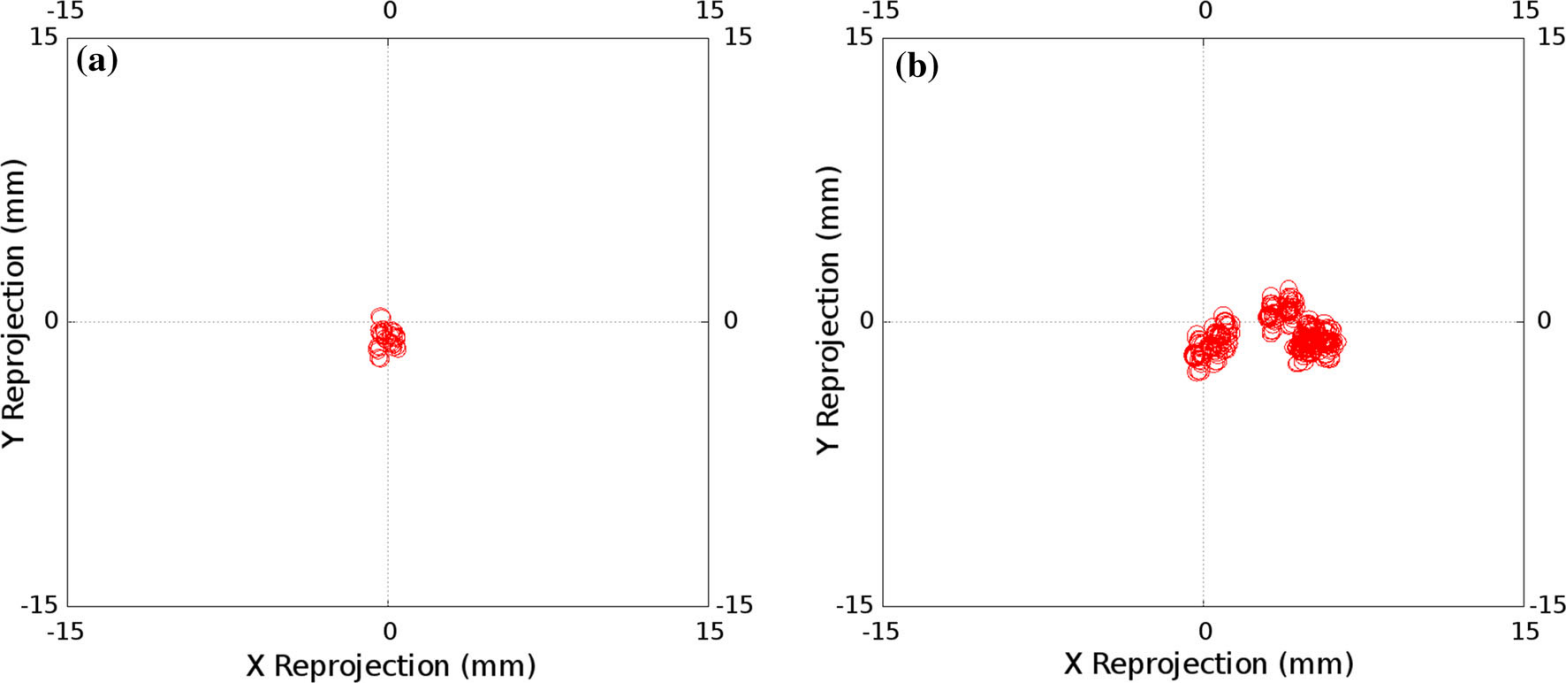
Projection errors for the calibrations performed using the chessboard using electromagnetic tracking. **a** Calibration using 30 chessboard images, with hand–eye calibration performed as per Tsai’s method; Ellipse Centre RMS = 1.00, Ellipse Mean Radii = 0.82. **b** Direct calibration, using a single image of a chessboard with known corner locations; Ellipse Centre RMS = 4.12, Ellipse Mean Radii = 0.88

Figure 8 shows the results of accuracy analysis of each of the invariant point calibration algorithms applied to the electromagnetically tracked laparoscope. Figure 9 shows the accuracy results using chessboard calibration for the electromagnetically tracked laparoscope. The same trend as seen for the optically tracked laparoscope is present, though in all cases the tracking and hand–eye calibration errors are significantly less.

## Discussion

The results presented in this paper show that the proposed calibration method is suitable for image-guided surgery applications. Different applications will have different requirements for hand–eye calibration accuracy and as such may adopt different optimisation approaches. It is clear that the optical tracking system has larger tracking errors at the lens due to the inability to place the markers near the tip. Therefore, calibration of the optically tracked system is in general more difficult. It is clear that being able to measure the location of the crosshair centre independently provides a significantly improved calibration for optical tracking, whereas the benefits are more marginal for an EM tracked system, provided the user checks for outliers.

A key advantage of the proposed system is the use of the projected sphere, as shown in Fig. 3, to enable real-time validation of the calibration result. The size of the sphere used will depend on the application. For the guidance system we are developing [25], accurate hand–eye calibration is not critical as the laparoscope is used for both localisation and visualisation, so inaccuracies in the calibration cancel out to some extent. In this case, we have been successfully using the system with a sphere of 10 mm radius. This accuracy can be achieved with optical tracking and an optimised invariant point location, as shown in Fig. 6a, b. However, should localisation of the liver be performed with a second, independently tracked probe, e.g. laparoscopic ultrasound [23], the hand–eye calibration errors directly effect overlay accuracy; hence, a sphere with a radius of 2 mm would be more appropriate. Electromagnetic tracking and a measured point location, as shown in Fig. 8c, d, would be necessary to achieve this accuracy.

The results presented for the optically tracked laparoscope appear to show that the method performs better than either Tsai’s method or direct solving using a chessboard. However, both these approaches are sensitive to the orientation of the views selected for calibration. Several authors have presented approaches to ensure that suitable views are used [19]; however, these can be difficult to implement in the surgical context. Our proposed method appears to be less sensitive to bias due to view selection.

We have shown that in our laboratory experiment, EM tracking results in smaller tracking errors and that hand–eye calibration using optical tracking can be very sensitive, producing larger tracking errors at the tip due to the lever effect. However, whereas we are currently able to use optically tracked scopes clinically on humans, we cannot yet use EM tracking at the tip due to requirements of sterility and robust attachment of the EM marker. Attaching an EM sensor to the distal end of the laparoscope would resolve line of sight issues, but the tracking accuracy of EM trackers is widely regarded as less accurate than optical trackers. Therefore, integration of EM trackers directly into surgical laparoscopes would obviously be of assistance to the development of image-guided laparoscopy, where the EM sensor must be placed near the tip.

The methods presented here were tested on a stereo laparoscope. Optimisation of the 3D reconstruction error requires a stereo laparoscope to enable triangulation of the on-screen points. The second cost function (projected errors) used will work on a monocular laparoscope as no triangulation is required. Optimisation using this cost function does, however, require a good initialisation to ensure convergence, so could only be used where the hand–eye calibration does not change significantly between procedures. This may be the case where the tracking collar is mounted with some sort of detent. Optimisation using projected errors appears to be slightly more accurate than optimisation using 3D reconstruction error. However, the results may be biased by the fact that our accuracy measurement is itself a projection error. Optimisation using projection errors will minimise errors parallel to the camera plane, at the expense of those normal to it, so may not be suitable in all applications.

The results presented in this paper provide a good indication of the effects on overlay accuracy of different hand–eye calibrations. While this is a measure of great importance for the intended application, it is not a direct measure of the accuracy of calibration. To measure the calibration accuracy directly requires that the ground truth calibration be known. This can be done via numerical simulation; however, the previous work [24] on numerical simulation of invariant point calibration has shown the critical importance of using error models that properly represent the tracking system. We intend to further study these and other calibration methods using realistic numerical simulations.

## Conclusion

This paper presents a method to perform hand–eye calibration of a laparoscope using an invariant point. The invariant point is defined as the centre of a crosshair. The advantages of our implementation are that it can be performed and evaluated in real time, by users with limited technical training. We have been using the system successfully in theatre. The results in this paper show that the system can perform as well or better than the most common existing methods. We have also compared optical (NDI Polaris Spectra) and electromagnetic (NDI Aurora) trackers and have demonstrated the benefits of placing an EM sensor at the camera end of the laparoscope.

## Footnotes

1 http://www.cascination.com.

2 http://www.pathnav.com.

3 http://www.vision.caltech.edu/bouguetj/calib_doc/.

4 http://www.opencv.org.
